# Measurements of Hazardous Air Pollutants in Communities Necessitate Review of Air Quality and Exposure Metrics

**DOI:** 10.1021/acs.estlett.6c00250

**Published:** 2026-09-28

**Authors:** Kelley C. Barsanti, Gabriel Isaacman-VanWertz

**Affiliations:** † Atmospheric Chemistry Observations and Modeling Lab, NSF National Center for Atmospheric Research, Boulder, Colorado 80301, United States; ‡ Department of Civil and Environmental Engineering, Virginia Tech, Blacksburg, Virginia 24060, United States

**Keywords:** air toxics, community monitoring, hazardous air pollutants, inhalation exposure

## Abstract

Advancements in observational capabilities have led to exponential increases in the number of compounds measured in air with unknown inhalation exposure guidelines or community health risks. Exposure frameworks try to capture a variety of exposure contexts with a range of guidelines; however, they are largely focused on occupational settings, and the relationship between guidelines is not always clear. Consequently, assessing measured compounds within these guidelines is not straightforward, and the question of whether air is safe is not easily answered. Here, the recommendations and aims of exposure frameworks are reviewed, which inherently drive guidelines and risk tolerance. U.S. and select international ambient air quality and inhalation exposure guidelines are compared, illustrating differences between the exposure frameworks and guidelines. For compounds with available exposure guidelines, this comparison provides order-of-magnitude estimates for protection of public health. To improve on these estimates, exposure frameworks are needed that build on ambient observations, target the most vulnerable community members, and consider real-world exposure durations from days to years.

## Graphical abstract

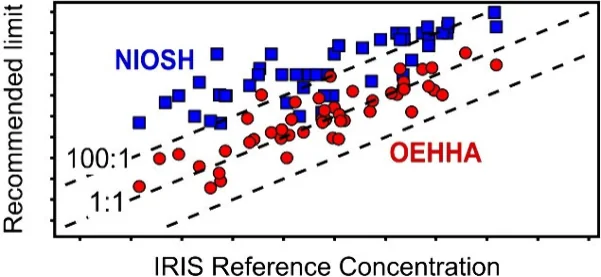

## 1 Introduction

Decades of advancements in instrumental and analytical techniques have led to increasing numbers of unique compounds being measured in outdoor air. Searching Web of Science yields at least 1,400 documents from the past 20 years that reference field measurements of the atmosphere, including hazardous air pollutants (HAPs). Within such studies, ∼10–20 organic compounds, including HAPs, with known public or environmental health concerns were measured routinely, while tens to hundreds were reported for short-duration measurement periods (i.e., days to weeks) with limited context for public or environmental health. A few recent example studies are highlighted here. Rico et al.^1^ reported measurements of over 100 organic compounds associated with the polluted Tijuana River in south San Diego County, California. Coggon et al. quantified hundreds of organic compounds associated with urban sources in New York City^2^ and Las Vegas.^3^ Permar et al.^4^ and Gkatzelis et al.^5^ reported measurements of over 100 organic compounds from wildland fires measured from aircraft. Many measured compounds can pose a health risk if they are present in sufficiently high quantities, but it is not always clear what quantities are sufficiently high to be of concern.^6^ Informing communities of risk and advising the public of safe exposure limits are challenging in the context of current ambient air quality frameworks and exposure guidelines that largely target occupational exposures.

### 1.1 Existing Regulatory Frameworks

In the context of ambient air quality, within the United States (U.S.), the National Ambient Air Quality Standards (NAAQS) set by the U.S. Environmental Protection Agency (EPA) are the only legally enforceable federal metrics for establishing whether outdoor air is “safe” for the public. The NAAQS define time-averaged concentration thresholds (typically based on 1, 8, 24 h, and/or annual averages) for only six criteria pollutants (carbon monoxide, lead, nitrogen dioxide, ozone, sulfur dioxide, and fine and coarse particulate matter), designed to be protective of the most sensitive populations assuming chronic (long-term, over a lifetime) exposure.^7^ The U.S. EPA also regulates 188 HAPs, 30 of which have been identified as posing the greatest public threat in urban areas and are therefore known as urban air toxics.^8^ However, these hazardous/toxic air pollutants are regulated at the source (i.e., emissions rather than ambient concentrations), so there are no explicit concentration thresholds for establishing legally enforceable safe or acceptable limits of these pollutants in outdoor air. Instead, the EPA and other state and federal scientific and regulatory bodies have established advisory exposure guidelines for outdoor levels, backed by a legally enforceable framework for occupational exposures. The origins of these guidelines are described in Section 1.2 and a subset is summarized in Section 3, including the EPA Acute Exposure Guideline Levels (AEGLs) and the Agency for Toxic Substances and Disease Registry (ATSDR) Minimum Risk Levels (MRLs).

Occupational exposure limits (OELs), due to their derivation and intended purpose, are reasonably higher than chronic exposure limits for sensitive populations (i.e., NAAQS). In the United States, the legally enforceable OELs come from the Occupational Safety and Health Administration (OSHA), which set Permissible Exposure Limits (PELs) in the 1970s around the same time the criteria and hazardous pollutant lists were established. These PELs account for chronic workplace exposure (e.g., 8 h work period, 40 h per week, over a lifetime).^9^ These limits, though still legally enforceable, are acknowledged to be outdated by OSHA, which instead proposes alternatives such as the Recommended Exposure Limits (RELs) set by the Center for Disease Control (CDC) National Institute for Occupational Safety and Health (NIOSH).^9^ The RELs establish threshold (averaged over a defined period of time) or ceiling (not to be exceeded) concentration limits in an 8 or 10 h work period. The California OHSA (Cal/OSHA) has also established an updated and extensive list of PELs that are enforceable within the state and provide guidance to other states.^10^

OELs are not easily translated to exposures experienced by the general public. This is in part due to the duration of exposure for which the limits were established, which is not necessarily the same for occupational workers as for the public. Furthermore, OELs are designed to protect healthy adult workers and not the most sensitive populations. Consequently, neither the NAAQS nor occupational-exposure based frameworks satisfactorily address outdoor concentrations safe for public exposures (chronic or otherwise) to HAPs. Instead, a number of advisory, but not legally binding, exposure guidelines have been established, with goals to protect workers and/or the public from acute exposure (short-term,with varying definitions up to 14 days) to outdoor air pollutants originating from, for example, a chemical spill. However, the advisory nature of these guidelines, the large number of state and federal agencies across which they have been established, and the increasing number of measured compounds with no such guidelines make it difficult to answer the seemingly straightforward question, “Is the air safe to breathe?”.

Here, we seek to contextualize the landscape of inhalation exposure limits and guidelines, particularly for and within communities where outdoor air measurements are being made. Environmental researchers collecting data may be tasked with interpreting those data in the context of public health risk, sometimes without the guidance of public health or risk experts. At the same time, public health and risk experts familiar with exposure limits and guidelines may not be aware of the rapidly expanding lists of compounds measured in the air and the disparate time scales of these measurements, real-world community exposures, and reference durations of exposure limits. Thus, we review and compare exposure limits and guidelines from a prominent subset of frameworks to help those without public health or risk expertise to better understand the intended purposes of these frameworks and the resultant wide ranges of guidelines, as well as to provide specific recommendations for using these frameworks in a public health risk context. Further, for public health and risk experts, we illustrate the need for informed communities and updated exposure frameworks.

While this review is focused on the U.S., we highlight a number of largely analogous frameworks in other countries. For example, in the context of ambient air quality standards, the Canadian Council of Ministers of the Environment has established the Canadian Ambient Air Quality Standards (CAAQs),^11^ the European Union (EU) has established air quality standards for the EU,^12^ and the Ministry of Ecology and Environment has established ambient air quality standards for China,^13^ each for a limited number of pollutants (up to 10) largely overlapping the six U.S. criteria pollutants. The World Health Organization similarly provides recommendations for the international community primarily for this same set of compounds.^14^ With these criteria pollutants, some countries include a small subset of HAPs regulated based on ambient concentrations, including Canada (arsenic, benzene, formaldehyde, and hydrogen sulfide)^15^ and the EU (benzene, arsenic, cadmium, nickel, and polycyclic aromatic hydrocarbons).^12^ However, like the United States, other countries largely regulate HAPs at the point of emission rather than by setting specific regulatorily binding ambient concentration thresholds.

Occupational exposure limit frameworks are also similar across countries. For instance, the EU requires member states to set OELs for a subset of HAPs. A recent review identified 1,400 compounds for which at least one member state set an OEL but notes there is no harmonized set across the EU.^16^ The German MAK commission led an effort to collate information through the GESTIS^17^ Substance Database, which contains chemical information as well as OELs for the EU and nine individual countries (including the U.S.) for ∼1,800 compounds. In Asia, the Japan Society for Occupational Health (JSOH) sets OELs for Japan, China’s National Health Commission oversees OELs set and enforced through several national and provincial entities,^18,19^ and the Republic of Korea’s OELs are overseen by the Ministry of Employment and Labor with a recent focus on increased enforcement.^20,21^ Exposure limits in an international occupational safety context have been previously reviewed.^22^ Here we seek to illustrate that, while the specifics of the frameworks vary, most countries, including the U.S., have established binding regulatory limits for occupational exposures, and there is an absence of such limits for ambient HAPs in a community-exposures context.

### 1.2 Inhalation Exposure Guidelines

Recognizing a need to develop guidelines for exposure to highly toxic compounds under nonroutine conditions (e.g., once in a lifetime), the U.S. EPA developed the Acute Exposure Guideline Levels (AEGLs) for Hazardous Substances. The AEGLs represent threshold limits for exposures ranging from 10 min to 8 h, and the intent has been to expand the list for which AEGLs exist to hundreds of compounds.^23^ There are three AEGL levels that represent increasing severity of effects, with AEGL-1 causing discomfort, AEGL-2 causing long-term damage or disability, and AEGL-3 causing potential death. The National Oceanic and Atmospheric Administration (NOAA) Office of Response and Restoration has stated that these AEGLs are appropriate for identifying hazards and protecting public health.^200^ However, we note the complexity in applying these guidelines for exposures in neighborhoods to chronic point sources (e.g., an industrial point pollution source), where a single 8 h exposure does not capture the true extent of exposure, and disability or death are not acceptable outcomes.

The Agency for Toxic Substances and Disease Registry (ATSDR), under the U.S. Department of Health and Human Services, is more specifically focused on community exposures. ATSDR has developed Minimum Risk Levels (MRLs), which are exposure guidelines for the general public below which there is no detectable risk for noncancerous health effects.^24^ MRLs have an advantage for public health risk in that they are established for acute (1–14 days), intermediate (15–364 days), and chronic (>364 days) exposures, though not all compounds have MRLs for all exposure durations (there are ∼175 total inhalation MRLs).^25^ At the state level, the California Office of Environmental Health Hazard Assessment (OEHHA) also sets RELs for public exposure to outdoor air pollutants for acute, 8 h, and chronic exposures.^26^ Both ATSDR and OEHHA guidelines state their goal is to protect sensitive populations from time-averaged and short-term exposures,^27,28^ and so reasonably they might be applied in a community-exposures context.

The U.S. EPA additionally maintains a database of toxicological data, the Integrated Risk Information System (IRIS), that provides information on toxicology or health risks of many compounds.^29^ The current EPA leadership has missed at least four releases of the triannual “IRIS Program Outlook”^30^ and has indicated an intention to eliminate this program,^31,32^ which would likely further hinder the ability to evaluate inhalation exposure health risks in communities. The IRIS data are provided here as Supplementary Data in part to assist in long-term data access. The IRIS database and similar frameworks use laboratory exposure studies and epidemiological data to assign an inhalation reference concentration (RfC) to each pollutant in the database.^33,34^ The details vary by pollutant, but generally the standardized approach relies on human/animal exposure studies to identify the level at which there is no or lowest observed adverse effect (NOAEL/LOAEL). These levels are then scaled to account for body weight and inhalation volume (standardized as 70 kg and 20 m^3^/day, with additional values available for subsets of the general population).^35^ Finally, the safe concentration is assumed to be lower by an uncertainty factor that attempts to account for uncertainties in the data (e.g., inter/intraspecies differences, sensitive populations, incomplete data). Within the IRIS database, uncertainty factors range from a minimum of 10 to a maximum of 3,000, set using a guideline of a factor of 3 or 10 for each source of uncertainty; most uncertainty factors are between 100 and 1,000. A similar toxicological approach is used for most sets of guidelines across agencies. For carcinogens, no safe level is assumed, and instead compounds are assigned an increased cancer risk per unit inhaled.^36^

An important aspect of the IRIS database in the context of community exposures is that the RfC was designed to be protective of sensitive populations under chronic exposure conditions. In this context, chronic refers to continuous exposure for a duration up to a lifetime, though it is practically considered by the U.S. EPA as “exposures greater than 10% of the human lifespan”.^36^ This is in contrast to occupational exposure frameworks, which as noted, were designed to be protective of healthy people in an occupational context (e.g., oil and gas refineries, wastewater treatment plants, etc.) under work day exposure conditions with appropriate levels of personal protection equipment. Differences in exposure conditions may warrant differences in uncertainty factors (e.g., whether or not to scale for sensitive populations) and thus may be expected to yield different exposure limits by an order of magnitude or more. The U.S. EPA has compiled information from many sources, including IRIS and AEGL, into the CompTox Chemicals Dashboard^37^ but does not in this effort seek to unify or explain differences between data sources and thus does not rectify the confusion that may be encountered when used by a broader community.

These databases of inhalation exposure guidelines for individual HAPs are relatively uncommon outside of the U.S., though we recognize that other databases may be available that are not in English and as such were not easily discoverable by the authors. Information on chemicals is compiled by several entities, for example, the European Chemicals Agency Registration, Evaluation, Authorisation and Restriction of Chemicals (ECHA/REACH) database, but these primarily maintain information on chemical risks, properties, and regulations rather than setting unified recommended exposure limits. Countries that do set national-level recommended exposure limits and are transparent about their methodology generally use a risk assessment framework similar to the IRIS database, incorporating toxicology and exposure modeling. For example, some member states of the EU follow this approach for a subset of compounds to set national thresholds for concern (e.g., Netherlands’ Environmental Risk Limits, which include various air, water, and soil guidelines), as does the United Kingdom, which makes publicly available Environmental Assessment Levels (EALs) for roughly one hundred compounds determined by the UK Environment Agency.^38,39^ Countries that lack national exposure guidelines may rely on lower-level governments; in Canada, for instance, roughly one hundred HAPs are regulated across six different provinces, in some cases with different recommendations between provinces.^40^ However, the number of national- or province-level entities with publicly available, easily accessible, and English-language databases of recommended exposure limits for hazardous compounds in ambient air is low,^38^ with most countries focused on water contamination and/or detailed reporting on a small number of compounds.

Recent work has pointed out the limitation of relying on individual risk levels such as RfCs for adequately assessing simultaneous exposure to multiple pollutants.^41^ Specifically, although a HAP may have adverse effects on multiple physiological systems, RfC levels are set at the threshold of concern for the most sensitive system. Traditional risk assessment is based on the sum of the ratios of measured concentrations to their RfCs, which may be based on different physiological systems. However, the sum exposure of multiple systems to multiple pollutants may exceed thresholds of concern, even in cases where a traditional risk assessment may not indicate concern. Nevertheless, although the current approach to risk assessment may need to be improved, the RfC from the IRIS database represents a current best estimate of toxicity for a wide range of HAPs with a highly transparent decision-making approach.^33^

### 1.3 Contrasting Exposure Guidelines and Regulatory Frameworks

To illustrate the differences in ambient air quality and occupational exposure metrics, we can look at standards and guidelines for the criteria pollutants carbon monoxide (CO) and sulfur dioxide (SO_2_) (Table 1). These are the only compounds for which an ambient outdoor concentration standard exists in the United States (NAAQS) and exposure limits are available (note that neither exist in the IRIS RfC database). It can be seen that recommended exposure limits are higher than NAAQS in all cases, usually by at least a factor of 2 and up to a factor of 4 to 25 for occupational exposure at longer time averages. Including the EU Air Quality Standards (AQS), EU OELs, and JSOH (scientifically recommended, although not binding) OELs support that these ambient air quality and occupational exposure frameworks are analogous globally. This comparison highlights that outdoor exposure limits for the general public should be set to be lower than occupational or emergency response limits. Using hydrogen sulfide (H_2_S) as an example, the IRIS RfC for this compound is 0.0014 ppm (1.4 ppb), which is approached only by the OEHHA chronic REL (7 ppb) and is at least an order of magnitude lower than any acute exposure limit. In particular, the limit set for a workplace context (i.e., NIOSH REL) is a very short-term limit and is more than one million times higher than any available chronic limits and more than ten thousand times higher than any limits designed for the general public. These comparisons demonstrate the range of applicable contexts across frameworks and illustrate that caution is needed when examining any outdoor air measurements in the context of inhalation exposures to sensitive populations over variable durations. The goal of this work is not only to review and intercompare widely used sets of guidelines but also to provide insight into the apparent goals and realistic contextual applications of each framework.

**Table 1:** Summary Intercomparison between Exposure Guidelines for Representative Compounds, Carbon Monoxide (CO), Sulfur Dioxide (SO_2_), and Hydrogen Sulfide (H_2_S)

	NAAQS	EUAQS	NIOSH REL^a^	OEHHA REL^a^	EU OEL	JSOH OEL-M	ATSDR MRL^a^	AEGL^b^
CO: carbon monoxide	9 ppm (8 h), once per year	8.7 ppm (8 h), daily max.	ceiling: 200 ppm	acu: 20 ppm	15 min: 100 ppm	8 h: 50 ppm	n/a	1 h: 83 ppm
			TWA: 35 ppm	chr: n/a	8 h: 20 ppm			8 h: 27 ppm
SO_2_: sulfur dioxide	0.075 ppm (1 h), 99th percentile	0.13 ppm (1 h), 0.05 ppm (24 h)	ceiling: 5 ppm	acu: 0.25 ppm	15 min: 1 ppm	n/a	acu: 0.01 ppm	1 h: 0.2 ppm
			TWA: 2 ppm	chr: n/a	8 h: 0.5 ppm		chr: n/a	8 h: 0.2 ppm
H_2_S: hydrogen sulfide	n/a	n/a	10 min: 10 ppm	acu: 0.03 ppm	15 min: 10 ppm	8 h: 5 ppm	acu: 0.07 ppm	1 h: 0.51 ppm
			TWA: n/a	chr: 0.007 ppm	8 h: 5 ppm		chr: n/a	8 h: 0.33 ppm

a Clarifications on time averaging: NIOSH limits use “ceiling” (generally <15 min) and “TWA” (time-weighted average, generally 8–10 h). OEHHA limits use “acute” (1 h) and “chronic” (1 year and longer). ATSDR limits use “acute” (1–14 days) and “chronic” (365 days and longer).

b The lowest available AEGL limit is listed, which is AEGL-1 for H_2_S and SO_2_ and AEGL-2 for CO.

## 2 Materials and Methods

Five exposure frameworks are reviewed in this work. For clarity, we use a single framework as a reference against which to compare the other four frameworks. We selected for this purpose the RfCs in the IRIS database. The stated intent of IRIS is to protect sensitive populations outside an occupational setting, suggesting that it is well-suited as a benchmark for community measurements, though we find it is not always the most conservative. Only compounds included in the IRIS database are shown in the figures, but data from all sources can be found in the Supplementary Data. The Supporting Information provides more detail about each data source, links to data sources, and the complete database of inhalation exposure guidelines from most data sources (for NIOSH, only compounds also found in IRIS are provided as Supplementary Data). The five sets of guidelines primarily examined here are shown in Table 2.

**Table 2:** Summary of Exposure Guidelines Examined in This Work

Framework/Agency	Guideline	Long-Term Duration^a^	Short-Term Duration^b^	Target Population
IRIS: EPA Integrated Risk Information System	Reference Concentration (RfC)	“chronic”	none	Sensitive populations
AEGL-1:^c^ EPA Acute Exposure Guideline Levels	Acute Exposure Guideline Level 1 (AEGL-1)	8 h	1 h	Intermittent or emergency exposures for the general public, including sensitive populations
NIOSH: CDC National Institute for Occupational Safety and Health	Recommended Exposure Levels (REL)	10 h: time-weighted average, “TWA”	10–15 min: “ceiling”	Occupational
ATSDR: CDC Agency for Toxic Substances and Disease Registry	Minimal Risk Levels (MRL)	365+ days: “chronic”	1–14 days: “acute”	General public
OEHHA: California Office of Environmental Health Hazard Assessment	Recommended Exposure Levels (REL)	“chronic”	1 h: “acute”	General public, including sensitive populations

a The longest exposure duration available was chosen from each framework for a long-term/chronic duration comparison.

b The shortest exposure duration closest to 1 h was chosen from each framework for short-term/acute duration comparison.

c AEGL-1 levels are used in this work where available, as they are the most conservative of the three levels and thus the most appropriate for community applications.

We focus primarily on guidelines that are publicly available and readily accessible in English, most of which originate in the U.S. However, in the Supporting Information, we also compare the United Kingdom EALs and Canada’s province-level guidelines against all five U.S.-based frameworks to examine how international guidelines may differ. Across these seven data sources, approximately 400 compounds have recommended inhalation exposure limits provided in the Supplementary Data; of these 400 compounds, the 92 that overlap with IRIS RfCs are included in the figures.

Though recommended levels are provided by agencies in a variety of units, all data are converted to ppb in this work; where necessary, mass concentration units are converted to ppb using molecular weights provided by the CAS number registry assuming an atmospheric pressure and temperature of 1 atm and 20 °C, respectively.

For all guidelines, the available limit for the longest term is used for “long-term”, which may be chronic (e.g., IRIS RfC) or may be only 8 h (e.g., AEGL). The available limit closest to a 1 h average is used for “short-term”, which may be as short as 15 min (e.g., NIOSH REL) or may be up to many days (e.g., ATSDR) and thus may be longer than the long-term limit from a different framework. This approach is necessary for examining the impact of time averaging on limits within each framework but also illustrates the inherent complexity of the exposure limit landscape and the need for updated frameworks that reflect exposures of communities under different real-world scenarios. For example, most OELs do not provide limits for exposure times longer than 8 to 10 h (a work day), so the available long-term limits within these frameworks are not likely to be appropriate for multiday or multiyear exposures. This expectation is confirmed by our analysis presented below and highlights the need for caution when applying these limits outside of their intended purpose, as well as the need for revised guidelines from public health and risk experts. This work does not include carcinogenic effects, as they are not included by some guidelines and are cataloged in separate databases for other guidelines. Carcinogens are presumed to have no safe lower limit and are assessed a potency factor rather than a recommended limit as examined in this work, so they should be considered separately in future work.

## 3 Results and Discussion

The primary results discussed in this review are intercomparisons between guidelines in order to understand the apparent aims and relative risk tolerance of each framework. Each set of guidelines has a stated target (e.g., occupational, sensitive populations, etc.), which may provide some context or interpretation of the recommended limits, but intercomparisons are used to evaluate conservativeness without a priori assumptions regarding intent. We do not seek to evaluate any specific toxicity or limit but rather assume that, as a rule, lower recommended limits are more appropriate for sensitive populations and community exposure contexts.

### 3.1 Comparisons of Long- and Shorter-Term Exposure Guidelines

When chronic, multiday exposure limits are available from a data source, as in the case of ATSDR and OEHHA, these values are generally similar to the IRIS RfCs (Figure 1). These two frameworks vary between an order of magnitude lower (more conservative) or higher (less conservative) than IRIS RfCs with a median of parity. IRIS, ATSDR, and OEHHA consequently form a cohort of comparable guidelines that may differ between specific compounds but are generally lower than other exposure limits and better-suited to assessing long-term risk to sensitive populations. In contrast, guidelines focused on an occupational context (NIOSH) or short-duration exposure for the general public (AEGL) are higher than the IRIS RfCs by 2 to 5 orders of magnitude (median: 2.5 and 3, respectively). For example, the chronic limit for H_2_S from OEHHA is within an order of magnitude of its RfC (5 times higher), while its AEGL-1 8 h limit is 230 times higher than the RfC. Other compounds like 1,2-dibromoethane (labeled in Figure 1) illustrate a more extreme example, with an OEHHA chronic limit 10 times lower than the RfC and an AEGL-1 8 h limit 4000 times higher than the RfC. As further discussed below, these two guidelines cannot be fully reconciled based on differences in reference exposure durations.

**Figure 1:**
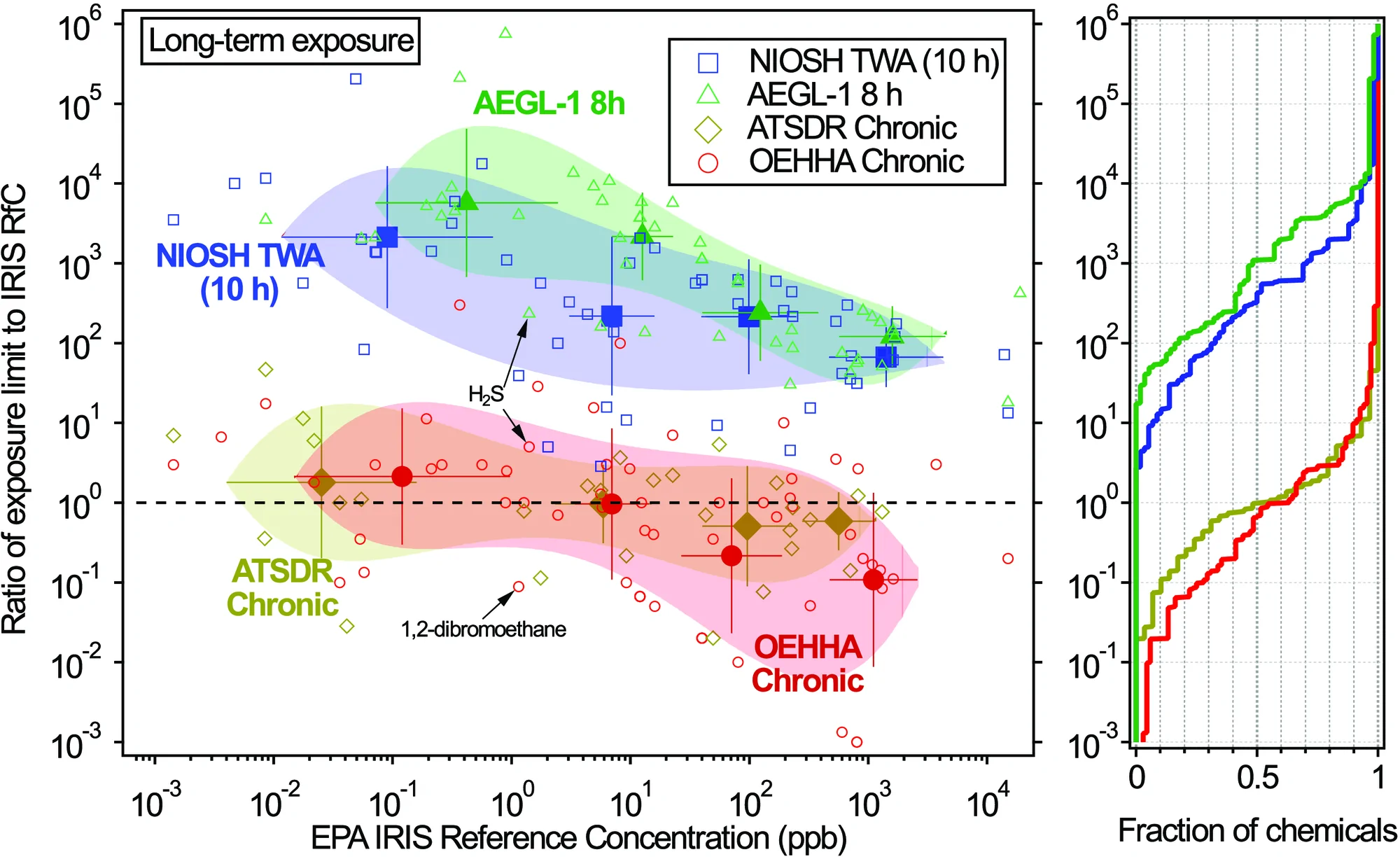
Comparison of long-term (range: 8 h to chronic) recommended exposure limits from each data source to the IRIS RfC as a function of IRIS RfC. Ratios of limits for individual compounds (i.e., limit in a given framework divided by the IRIS RfC) are open symbols. Filled symbols are quartile averages with error bars showing the standard deviation in each quartile, approximately outlined by the shaded region. The dashed line at 10^0^ defines parity between a limit and the IRIS RfC. The right panel shows the cumulative distribution of ratios for each data source using the same colors.

The relatively high long-term exposure limits for the AEGL-1 and NIOSH REL (TWA) guidelines may be due in part to their shorter reference duration than ATSDR and OEHHA guidelines (chronic), with the longest available averaging times of only 8–10 h. This effect of time averaging can be qualified, in part, by comparing short-term exposure limits (Figure 2). The exposure limits closest to 1 h were selected, though they vary across frameworks: ATSDR “acute”, OEHHA “acute”, and AEGL are 1–14 days, ∼1 h, and 1 h, respectively; NIOSH short-term varies subhourly (typically 10–15 min). Relative to long-term limits, short-term median limits from ATSDR and OEHHA increase by approximately an order of magnitude, while AEGL and NIOSH increase by only a factor of ∼2. Short-term ATSDR and OEHHA limits are still more conservative than AEGL and NIOSH, though the short-term limits are somewhat closer together. These data reflect the fact that dosages are often time averaged, so a higher-level exposure for a shorter duration yields a similar average dose and thus assumed adverse effect to a lower-level exposure for a longer duration. This assumption is generally a consensus across frameworks and is included explicitly in some frameworks that provide multiple time averages, such as the AEGL. However, the relative differences across frameworks are not explained by time averaging alone. When comparing the OEHHA and AEGL-1 1 h exposure duration, OEHHA limits are several of orders of magnitude lower. As an example, in comparison to the RfC (chronic), 1 h exposure limits for H_2_S from OEHHA and AEGL-1 are 20 and 500 times higher, respectively, despite having the same time averaging and both seeking to protect sensitive populations. To put this in another perspective, if we think about time-averaged exposure, every hour of exposure at the AEGL-1 limit would be equivalent to ∼25 h of exposure at the OEHHA limit. This discrepancy leaves community members with little guidance in selecting the most appropriate safe limit.

**Figure 2:**
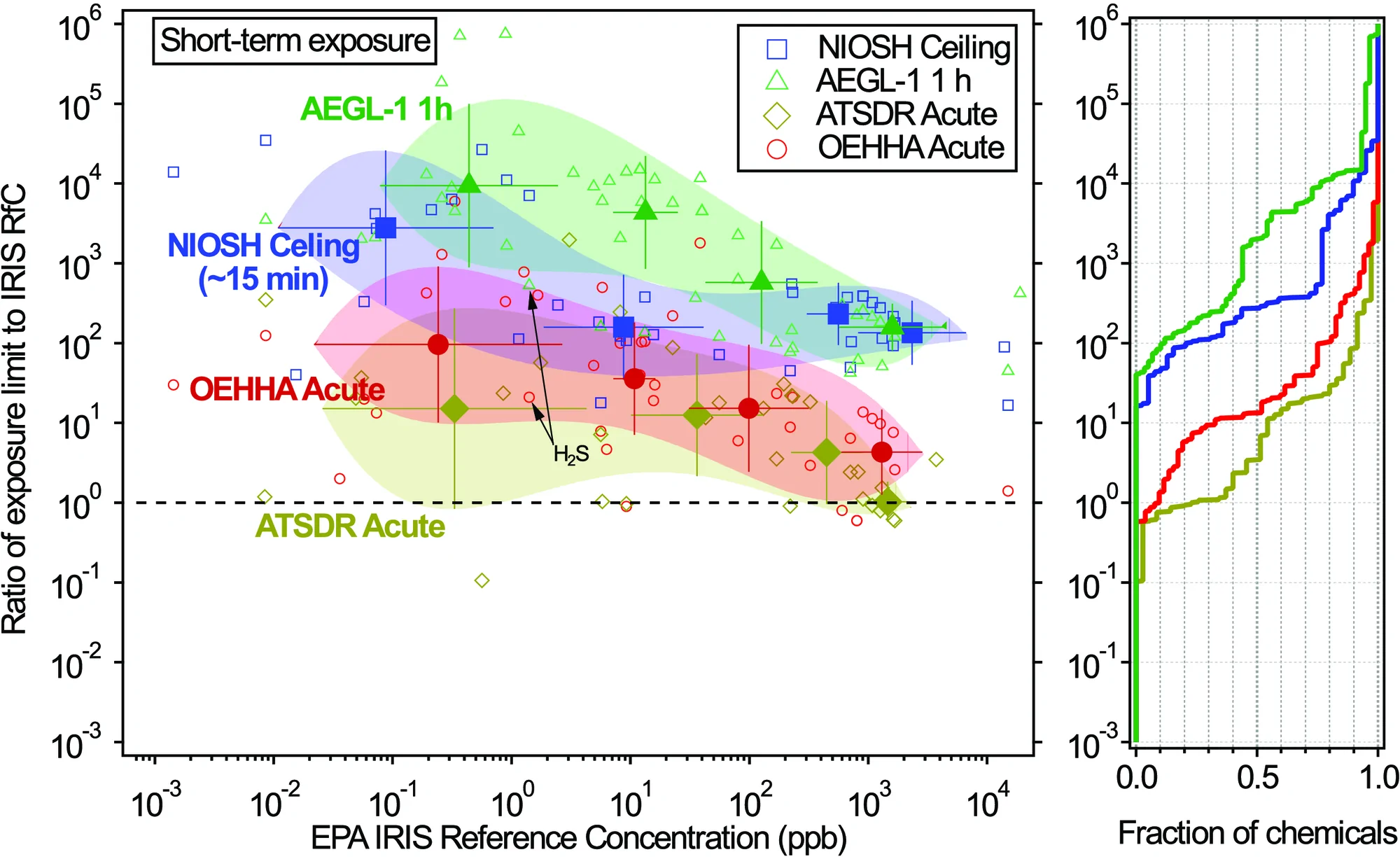
Comparison of short-term (range: 15 min to 1–14 days) recommended exposure limits from each data source to the IRIS RfC as a function of IRIS RfC. Ratios of limits for individual compounds (i.e., limit in a given framework divided by the IRIS RfC) are open symbols. Filled symbols are quartile averages with error bars showing the standard deviation in each quartile, approximately outlined by the shaded region. The dashed line at 10^0^ defines parity between a limit and the IRIS RfC. The right panel shows the cumulative distribution of ratios for each data source using the same colors.

### 3.2 Utility of Each Framework in the Context of Community Applications

Together, the long- and short-term limits of each framework support that ATSDR, OEHHA, and IRIS were derived for similar purposes (chronic exposures to sensitive populations) with comparable limits when accounting for differences in the exposure duration. Like IRIS RfCs, the OEHHA and ATSDR focus on general and sensitive populations. The (English-language) international guidelines identified in this review from the United Kingdom and Canadian provinces generally follow the IRIS RfC for long-term exposures and the ATSDR and OEHHA exposure guidelines for both shorter- and longer-term exposures (Figures S1 and S2). Each data source includes information about different sets of compounds, so community members or researchers may find relevant guidelines in one or more of these databases. Our analysis suggests that any guideline in one of these data sources can be viewed as approximately comparable to the others and that these data sources achieve similar aims.

AEGL and NIOSH limits are more permissive, and to some extent, this is reflective of different aims. NIOSH addresses an occupational context for which uncertainty factors used in the limit-setting determination might reasonably be 10–100 times lower (e.g., excluding intraspecies uncertainty factor), and thus limits of 10–100 times higher are expected. Tabulated values are somewhat higher than this expectation, with NIOSH guidelines on average a factor of 300 higher than chronic guidelines such as RfC. A combination of shorter-term exposures and excluded uncertainty factors can approximately explain these differences but highlight the limitation of these guidelines for providing risk or health guidance relevant for longer-term exposure by sensitive populations. The decision-making priorities of AEGL appear to be more complex, as they are “designed to protect the elderly and children, and other individuals who may be susceptible”^42^ but are more permissive than other frameworks with similar stated goals. This framework was created to manage acute chemical releases and so may reflect a goal of damage mitigation in an emergency context. Overall, these data suggest that neither NIOSH nor AEGL limits should be strictly applied to assess chronic or intermittent pollution in a community (e.g., from a local industrial source), but they may be appropriate in other contexts such as occupational exposure.

One somewhat surprising observation is the systematic impact of toxicity, as assessed by IRIS, on permissiveness of each guideline. For all frameworks, both long- and short-term, the ratio of each exposure limit to the RfC is lower for the least toxic compounds (highest RfC) than for the most toxic. This is statistically significant for all data sets (*p* < 0.05 using a Wilcoxon–​Mann–Whitney rank test with no assumption of normality), except long-term ATSDR limits which are generally similar to RfC. For OEHHA, this trend actually means that long-term limits for less toxic compounds are more conservative than IRIS RfCs, and they are brought to parity for more toxic compounds. However, for AEGL and NIOSH, less toxic compounds have limits 100 times higher than IRIS RfCs, while more toxic compounds have limits 1,000–10,000 times higher. Put simply, these trends mean that for many guidelines, including occupational limits, recommended limits tend to be relatively more stringent for less toxic compounds than for more toxic compounds. For less toxic compounds, a factor of ∼100 difference as is observed for comparisons between occupational limits and IRIS RfCs can be easily explained by differences in target populations and thus uncertainty factors. The large disparity for more toxic compounds may indicate different priorities, roles of other stakeholders, or differences in toxicology data used in decision-making. It may also represent limitations in technologies at the time of decision-making, as some frameworks (particularly those that are legally enforceable) may have chosen to set exposure limits at or near the limits of detection based on measurement capabilities, while in contrast there is no explicit consideration of the feasibility of achieving or measuring a limit in setting RfCs. Likely these trends reflect some combination of these factors, but a causal explanation is not possible from the data alone.

## 4 Recommendations and Research Needs

The review and analysis of existing exposure limits and guidelines suggest that the best way to protect public health is to establish unified and binding community exposure guidelines for the wide range of known HAPs/air toxics over a wide range of exposure durations that are protective of public health. Such guidelines ideally would be codeveloped by communities, researchers, and public health and risk experts, such that they are easily interpretable and reflect the ranges of compounds measured in outdoor air, both levels and time averages, and the associated exposure durations likely encountered by communities. Until such a framework exists, community inhalation exposures can be contextualized by understanding the intent (both stated and apparent) of existing exposure frameworks and comparing existing exposure guidelines, as done herein. These existing frameworks also provide guidance for scaling occupational exposure or other less conservative limits to be protective of public health.

The right panels in Figures 1 and 2 show cumulative distribution plots of the differences (as ratios) between the IRIS RfC exposure limits and those of other frameworks. These distributions were used to derive conversion factors to scale each guideline to IRIS RfC values, provided in Table 3. Because there is some spread in the differences, there is necessarily some range in the conversion factors, which can be considered in the context of risk tolerance; risk-averse conversion factors would reduce a larger fraction of compounds in a framework to the IRIS RfC or lower, while risk-tolerant factors would leave a larger fraction of compounds in a framework still above the IRIS RfC. The values in Table 3 demonstrate that ATSDR and OEHHA chronic values are generally in line with IRIS toxicological data and likely applicable to community exposure contexts. In contrast, NIOSH and AEGL tend to be 1 to 4 orders of magnitude higher.

**Table 3:** Multiplicative Factors That Statistically Bring Each Guideline Down to the IRIS RfC for Compounds Contained in Both Data Sets^a^

To Bring Guideline Down to IRIS RfC or Lower, Divide by			
	Risk-Averse	Median	Risk-Tolerant
AEGL-1			
1 h	20,000	2,000	80
8 h	10,000	1,000	50
NIOSH REL			
Ceiling	10,000	300	50
Time-weighted average	3,000	300	10
ATSDR MRL			
Acute (<14 days)	80	3	0.8
Chronic (>1 year)	5	1	0.1
OEHHA REL			
Acute (1 h)	400	10	1
Chronic (>1 year)	10	0.6	0.02

a Risk-averse brings 90% of values down to IRIS RfC or lower, median 50%, and risk-tolerant 10%.

The application of the conversion factors in Table 3 provides approximate limits for chronic community exposure when limits from one or more of these frameworks are available. As an example, piperidine has an AEGL-1 8 h limit (1.7 ppm) but no guidance from ATSDR or OEHHA or an IRIS RfC. In the case of ongoing community exposure, none of the frameworks reviewed in this work would be appropriate; instead, a concentration in the range of 100–10,000 times lower than the AEGL-1 limit might be considered a reasonably safe threshold. The AEGL-1 8 h limit (1.7 ppm) is divided by 1,000 to bring it in line with IRIS RfC under median risk tolerance; this results in an approximate limit of 1.7 ppb for long-term community exposure. International guidelines, at least including those English-language data sources examined here, can also provide an additional avenue for potential exposure recommendations for communities, as they approximately follow the more conservative U.S.-based frameworks; in some cases, they may even be systematically more conservative, as in the case of guidelines provided by Quebec (Figure S3).

We conclude with four recommendations. First, there is a clear need to combine exposure limits and underlying data across frameworks. Recent work has made progress in this direction by, for example, combining data from IRIS, ATSDR, and others into CompTox, which significantly advances this recommendation but does not yet include all compounds included across the many databases. Furthermore, while bringing together these disparate frameworks is useful, it does not assist nonexperts in understanding the differences between guidelines or reconciling cases in which frameworks with similar stated aims differ substantially in guidelines. Thus, second, for compounds that exist in one or more databases, risk expertise is needed to develop consensus or unified guidelines for real-world exposure durations protective of community health. As an example, propylene has no IRIS RfC or ATSDR MRL, so it does not have a CompTox entry for chronic exposure despite having an OEHHA REL. Similarly, compounds like H_2_S for which OELs exist have enough available toxicity data to be included in some state- or provincial-level limits (e.g., OEHHA) but are not included in many national (e.g., U.S. or EU) frameworks. A consensus framework, particularly one with regulatory power in one or more countries, represents a substantial challenge of cooperation but would provide the strongest protections for community exposure. Third, we recognize that for some compounds there is insufficient toxicological data to adequately assess risk and therefore recommend that the continued generation of such data focuses in particular on the context of chronic exposures whenever possible. Lastly, while it is not reasonable for environmental scientists and researchers to also be experts in public health and risk assessment, we strongly recommend that researchers working within communities be aware of the landscape of guidelines in which their measurements are being collected. Until the establishment of unified guidelines or enforceable limits for community exposures to air toxics, it is critical that measurements are placed in the context of an appropriate set of guidelines given the very wide range of “acceptable” exposure limits.

## Supplementary Material





## Data Availability

All data used in this work are provided as Supplementary Data.

## Notes

Supplementary Data: Includes short- and long-term exposure guidelines for all available compounds from all seven data sources (XLSX)
Supporting Information: Additional details of the five U.S.-based data sources and two additional English-language international data sources, as well as figures comparing international data sources to each other and to U.S.-based data sources (PDF)
