# Supplementary material for: Measurements of Hazardous Air Pollutants in Communities Necessitate Review of Air Quality and Exposure Metrics
Source: Environ Sci Technol Lett. 2026 Sep 28;13(10):1280–8. doi: 10.1021/acs.estlett.6c00250 (PMC13671167; doi:10.1021/acs.estlett.6c00250)
Supplement: Supplementary file 1 [file ez6c00250_si_002.pdf]

# Supplementary Information: Measurements of Hazardous Air Pollutants in Communities Necessitate Review of Air Quality and Exposure Metrics

Kelley C. Barsanti[1†], Gabriel Isaacman-VanWertz[2†*]

[1]Atmospheric Chemistry Observations and Modeling Lab, NSF National Center for Atmospheric Research, Boulder, CO 80301

[2] Department of Civil and Environmental Engineering, Virginia Tech, Blacksburg, VA 24060

[†]contributed equally as authors

*address correspondence to: ivw@vt.edu

## S1. Detail regarding data sources

The five U.S.-based data sources are:

(1) US EPA IRIS database Reference Concentrations (RfCs). Values used in this work are downloaded directly from iris.epa.gov (last accessed December 29, 2025). RfCs are determined for chronic exposure and are set for sensitive populations. For every compound, a detailed report is available for how the RfC is set. The database may contain multiple RfCs for each unique compound, set for different adverse effects; all RfCs are included in this work, so a compound may be represented by multiple data points.

(2) US EPA Acute Exposure Guideline Levels (AEGLs). Values used in this work are provided by the EPA in the document "Combined AEGL Values" updated July 27, 2018, accessible at https://www.epa.gov/aegl/compiled-acute-exposure-guideline-values-aegls (last accessed December 29, 2025). AEGLs are determined for 3 levels representing severity of symptoms, for which a guidance document is publicly provided and can be broadly classified as: AEGL-1 - discomfort, AEGL-2 - disability, AEGL-3 - death. Only AEGL-1 levels are used in this work where available. Levels are published for 6 different time averages, from 10 minutes to 8 hours, for which the calculation approach is provided in the guidance documents. In this work, the longest available time average (8 hours) is used for longer-term exposure, and the 1-hour time average is used for shorter-term exposures. Detailed information related to decision making is available for each compound individually.

(3) CDC NIOSH Recommended Exposure Limits (RELs). Values used in this work are from the NIOSH Pocket Guide, which has been made digitally accessible as described in Lucas et al. (2024); because of this publicly accessible database, only the guidelines for compounds also in the IRIS database are reproduced in the Supplementary Data. As described above, NIOSH RELs are determined for an occupational context. RELs for compounds are determined for a time-weighted average (TWA, typically assuming 10 hours) or shorter term exposures (typically 15 minutes, but variable by compound) which may be provided as either a short term exposure limits (STEL) or a ceiling REL; these two are taken as equivalent in this work and used as a short term REL. Data is included in this work for both TWA RELs

and short term RELs where available, though not all compounds have both. Exposure studies used to set levels are provided for most compounds, but while criteria documents describe decision making for some compounds, clear information is not always available at the compound level.

(4) CDC Agency for Toxic Substances and Disease Registry (ATSDR) Minimal Risk Levels (MRLs). Values used in this work are directly from the CDC website at https://wwwn.cdc.gov/TSP/MRLS/mrlsListing.aspx (last accessed December 29, 2025), published in the document "MINIMAL RISK LEVELS (MRLs)", with the edition published May 2025 used in this work. MRLs are determined for three different averaging times including acute (1-14 days), intermediate (>14-364 days), and chronic (365 days and longer). In this work chronic is used for long-term exposure and acute is used for shorter-term exposure. We note the complexity in this terminology and intercomparison, as the short-term exposure period for ATSDR MRLs is actually longer than the longest-term exposure period of NIOSH RELs and EPA AEGLs. Detailed information regarding how MRLs are determined are provided for every compound.

(5) California Office of Environmental Health Hazard Assessment (OEHHA) Recommended Exposure Levels (RELs). Values used in this work are directly from the OEHHA website at https://oehha.ca.gov/air/general-info/oehha-acute-8-hour-and-chronic-reference-exposure-level-rel-summary (last accessed December 29, 2025), copied directly from the website, with an update date of October 11, 2023. MRLs are determined for three different averaging times including acute (maximum 1 hour), 8-hour, and chronic. In this work chronic is used for long-term exposure and acute is used for shorter-term exposure. A high level of detail is provided in the publicly available guidance document about how health risk assessment is conducted and sample information about some compounds. The method used for every compound is provided as a technical support document.

All acute inhalation data for all five data sources are provided as Supplementary Data. Cancer risk in not examined in this work.

In addition, two English-language international data sources are included in Supplementary Data:

(1) United Kingdom Environmental Assessment Levels. Values used in this work are directly from the UK Environment Agency website at https://www.gov.uk/guidance/air-emissions-risk-assessment-for-your-environmental-permit (last accessed March 10, 2026), copied directly from the website. Levels are reported for a variety of averaging times, with averaging times one day or longer used for long-term exposure and one hour or shorter used for shorter-term exposure.

(2) Canadian provincial exposure levels. Previous work by Galarneau et al. (2015, main text ref. 40) compiled recommended inhalation exposure levels from six Canadian provinces, and these data are included here. Levels are reported for a variety of averaging times, with averaging times one day or longer used for long-term exposure and one hour or shorter used for shorter-term exposure. Where multiple short- or long-term averaging times for one compound are provided, the longest is used.

S2. Comparisons for international data sources

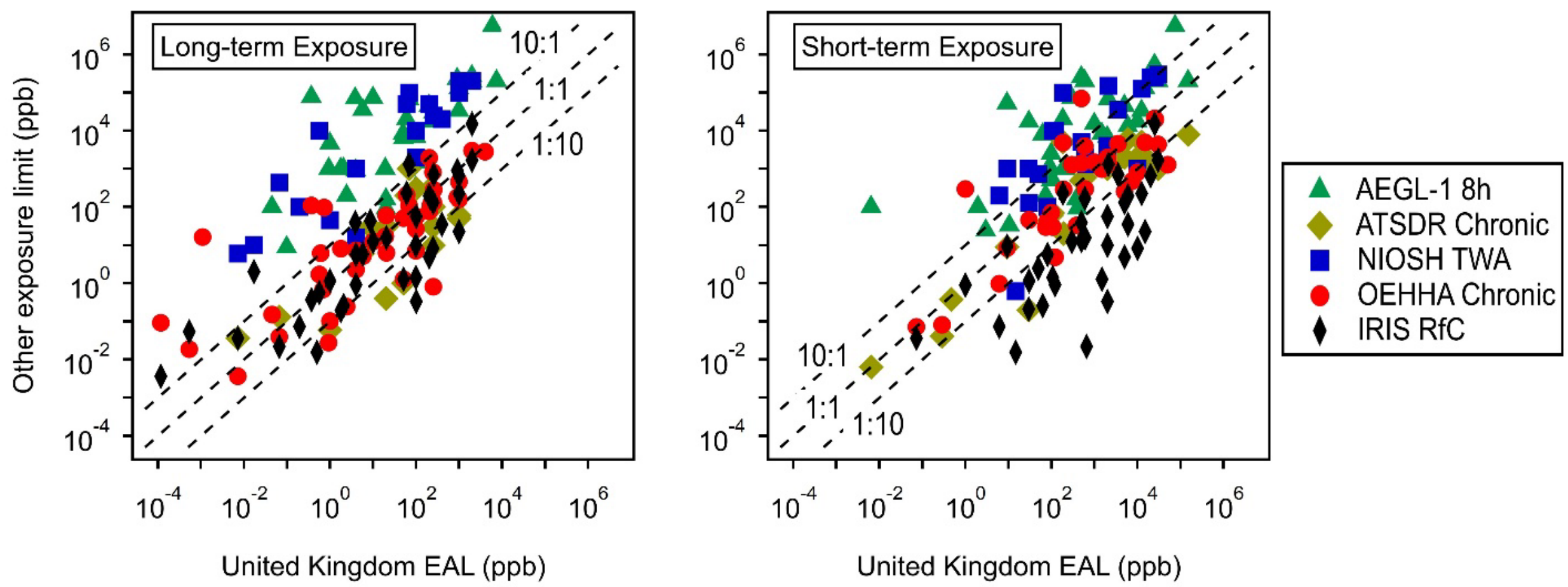


Figure S1. Comparison between all U.S.-based recommended exposure limits and the United Kingdom Environmental Assessment Levels, for (left) long-term and (right) short term exposures. Colors and symbols are the same as in Figures 1 and 2, with IRIS RfC added as place diamonds.

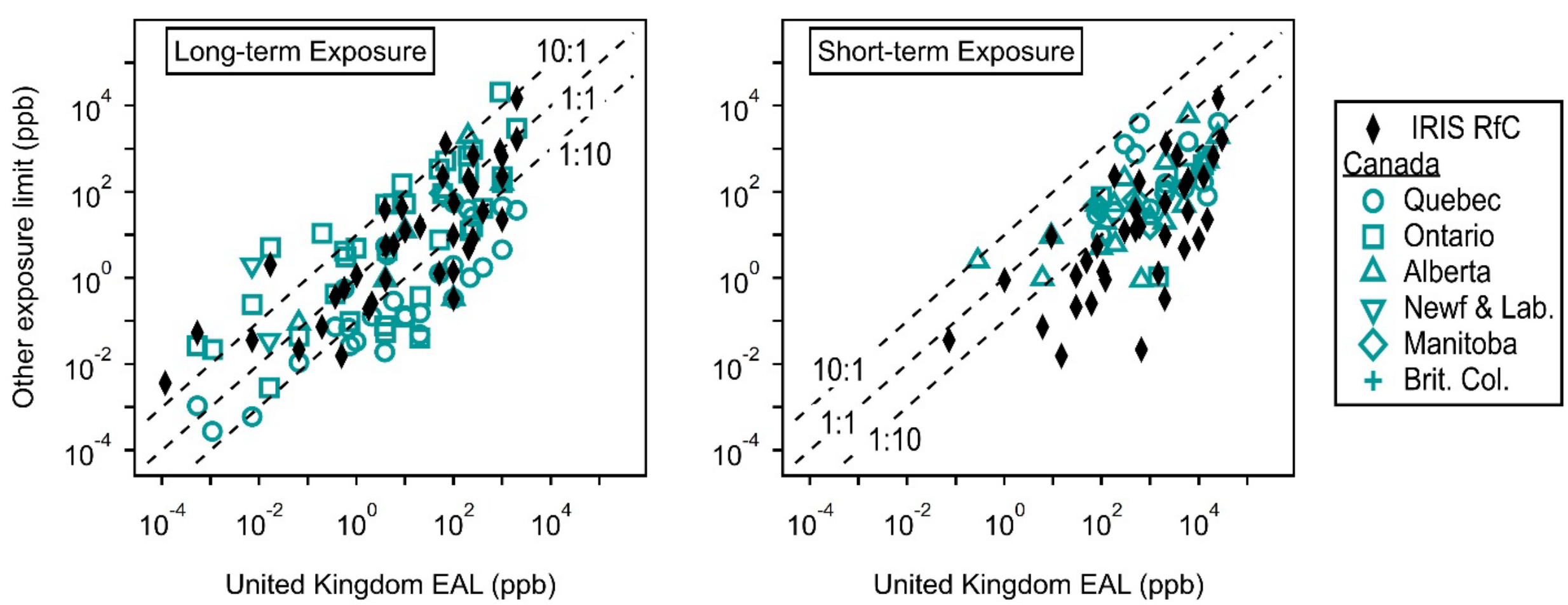


Figure S2. Comparison between international recommended exposure limits, specifically Canadian provincial limits against United Kingdom Environmental Assessment Levels with IRIS RfC for comparison, for (left) long-term and (right) short term exposures. Empty symbols represent recommendations from different provinces, with most limits form Quebec, Ontario, and Alberta.

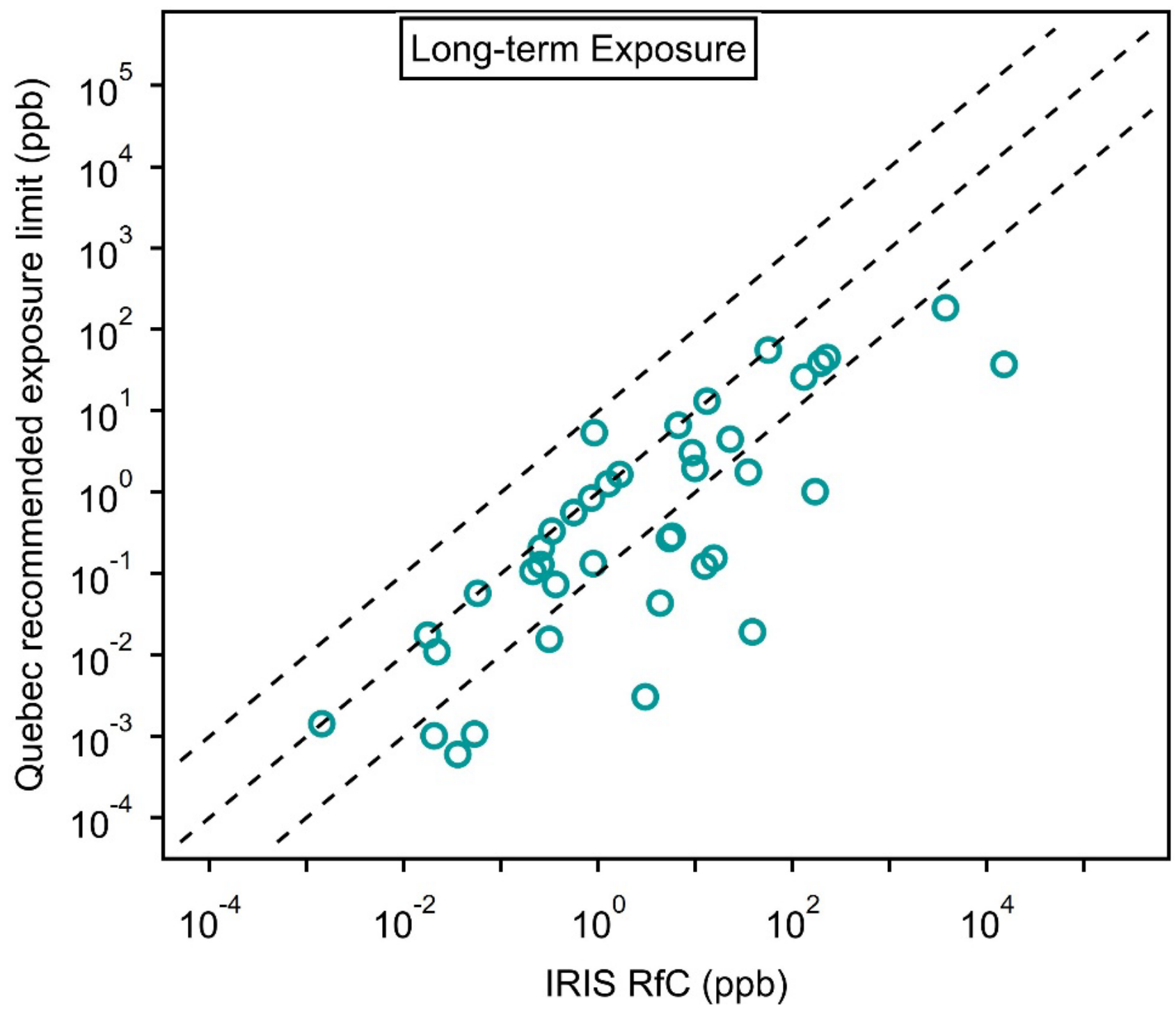


Figure S3. Comparison between recommended exposure limits from Quebec and IRIS RfC demonstrating more conservative limits set by the Canadian province.
